# Previous *Helicobacter pylori* infection–induced atrophic gastritis*:* A distinct disease entity in an understudied population without a history of eradication

**DOI:** 10.1111/hel.12669

**Published:** 2019-11-03

**Authors:** Hiroshi Kishikawa, Keisuke Ojiro, Kenji Nakamura, Tadashi Katayama, Kyoko Arahata, Sakiko Takarabe, Soichiro Miura, Takanori Kanai, Jiro Nishida

**Affiliations:** ^1^ Department of Gastroenterology Ichikawa General Hospital Tokyo Dental College Ichikawa Chiba Japan; ^2^ Graduate School International University of Health and Welfare Minato‐ku Tokyo Japan; ^3^ Department of Internal Medicine Division of Gastroenterology and Hepatology Keio University Shinjyuku‐ku Tokyo Japan

**Keywords:** chronic atrophic gastritis, endoscopy, eradication, gastric autoimmune diseases, *Helicobacter pylori* diagnosis, *Helicobacter pylori* infection

## Abstract

Individuals with chronic atrophic gastritis who are negative for active *H. pylori* infection with no history of eradication therapy have been identified in clinical practice. By excluding false‐negative and autoimmune gastritis cases, it can be surmised that most of these patients have experienced unintentional eradication of *H. pylori* after antibiotic treatment for other infectious disease, unreported successful eradication, or *H. pylori* that spontaneously disappeared. These patients are considered to have previous *H. pylori* infection–induced atrophic gastritis. In this work, we define these cases based on the following criteria: absence of previous *H. pylori* eradication; atrophic changes on endoscopy or histologic confirmation of glandular atrophy; negative for a current *H. pylori* infection diagnosed in the absence of proton‐pump inhibitors or antibiotics; and absence of localized corpus atrophy, positivity for autoantibodies, or characteristic histologic findings suggestive of autoimmune gastritis. The risk of developing gastric cancer depends on the atrophic grade. The reported rate of developing gastric cancer is 0.31%‐0.62% per year for successfully eradicated severely atrophic cases (pathophysiologically equal to unintentionally eradicated cases and unreported eradicated cases), and 0.53%‐0.87% per year for spontaneously resolved cases due to severe atrophy. Therefore, for previous *H. pylori* infection–induced atrophic gastritis cases, we recommend endoscopic surveillance every 3 years for high‐risk patients, including those with endoscopically severe atrophy or intestinal metaplasia. Because of the difficulty involved in the endoscopic diagnosis of gastric cancer in cases of previous infection, appropriate monitoring of the high‐risk subgroup of this understudied population is especially important.

## 1 INTRODUCTION

Since the International Agency for Research and Cancer (IARC) of the World Health Organization designated *H. pylori* a type 1 carcinogen in 1993,^1^ *H. pylori* infection has been widely accepted as the strongest risk factor for the development of gastric cancer, and numerous studies have supported this association.^2^, ^3^, ^4^, ^5^ The high prevalence of gastric cancer in *H. pylori*‐positive subjects likely occurs because *H. pylori* infection leads to the progression of chronic atrophic gastritis with intestinal dysplasia, which significantly increases the risk of gastric cancer.^6^ Eradication of *H. pylori* can be an effective method of treatment for peptic ulcer disease^7^ and mucosa‐associated lymphoid tissue lymphoma.^8^ Furthermore, eradication is especially important for reducing the development of new‐onset gastric cancer^3^, ^9^, ^10^ as well as secondary gastric cancer after endoscopic treatment. 4, 11, 12 Therefore, eradication of *H. pylori* has been used globally for approximately 30 years. In 2014, the IARC recommended population‐based screening and eradication of *H. pylori*, if feasible, because *H. pylori* causes 90% of non‐cardia cancers, and a 30%‐40% reduction in the incidence of gastric cancer is expected with the use of eradication therapy.^13^

Several investigators have reported that a certain percentage of subjects, excluding false‐negative cases and post‐eradication cases, showed endoscopic or histologic atrophy without a current *H. pylori* infection. A similar subpopulation has also been recognized in Japan, with patients showing atrophic gastritis endoscopically despite serologically normal gastric cancer screening using a pepsinogen (PG) and *H. pylori* antibody titer (ie, the ABC method).^14^, ^15^, ^16^, ^17^, ^18^ Plausible explanation for this phenomenon includes the spontaneous elimination of *H. pylori* because of the following: unintentional *H. pylori* eradication treatment, which could occur after exposure to antibiotics for the treatment of another infection; spontaneous disappearance of *H. pylori* as a result of severe atrophy; or previous administration of eradication treatment that patients had forgotten. Another explanation for this phenomenon could be autoimmune gastritis. However, it is important to note that compared with autoimmune gastritis, the spontaneous elimination of *H. pylori* is a distinct disease entity in the sense that the development of gastritis originates from *H. pylori* despite patients being negative for the presence of *H. pylori* infection. Clinicians should be aware of this distinction.

Herein, we highlight these previous *H. pylori* infection–induced atrophic gastritis cases, especially because this subpopulation is at high risk of gastric carcinogenesis despite their *H. pylori*‐negative infection status. To date, only a few investigators have focused on these subjects.^14^, ^15^, ^16^, ^17^

In this review, we describe the disease entity, definition, epidemiology, and serologic characteristics of these subjects. Furthermore, we propose an optimal endoscopic surveillance interval for such patients.

## 2 DEFINITION OF PREVIOUS *H. pylori* INFECTION–INDUCED ATROPHIC GASTRITIS

To date, only Hiyama et al defined unintended eradication, which is similar to our definition of disease entity as negative results of three *H. pylori* tests; the presence of glandular atrophy according to histologic examination; and no medical history of *H. pylori* treatment. However, autoimmune gastritis was found during their analysis, even though they did not specifically discuss these conditions.^14^

When defining previous *H. pylori* infection–induced atrophic gastritis, we aim for a simple diagnosis based on the results of *H. pylori* tests, a medical examination, and endoscopic findings during daily clinical practice; diagnostic assistance using histology and specific serologic examination were necessary in some circumstances. We defined the criteria for unintended elimination of *H. pylori* as follows: absence of a medical history of specific *H. pylori* eradication therapy; atrophic changes according to endoscopy or histologic diagnosis of glandular atrophy; absence of endoscopically localized corpus atrophy or positive autoantibody or characteristic histology suggestive of autoimmune gastritis; and negative for a current *H. pylori* infection. These criteria are detailed in Table 1.

**Table 1:** Practical criteria to diagnose previous *H. pylori* infection–induced atrophic gastritis cases

Condition	Criteria
Past history of *H. pylori* eradication	No past history of *H. pylori* eradication
Diagnosis of mucosal atrophy	Endoscopically atrophic changes more than C2 in the Kimura‐Takemoto classification or glandular atrophy on histology
Exclusion of rare types of gastritis unrelated to *H. pylori* infection	Exclusion of autoimmune gastritis by endoscopic findings regarding the distribution of atrophy or by autoantibodies or histology
Diagnosis of negative for present *H. pylori* infection	Negative results for the urea breath test or stool antigen test while patient is not using PPIs and antibiotics. Positive serology with negative urea breath test or stool antigen test strongly suggests past infection but absence of infection presently.

Abbreviation: PPIs, proton‐pump inhibitors.

**Figure 1:**
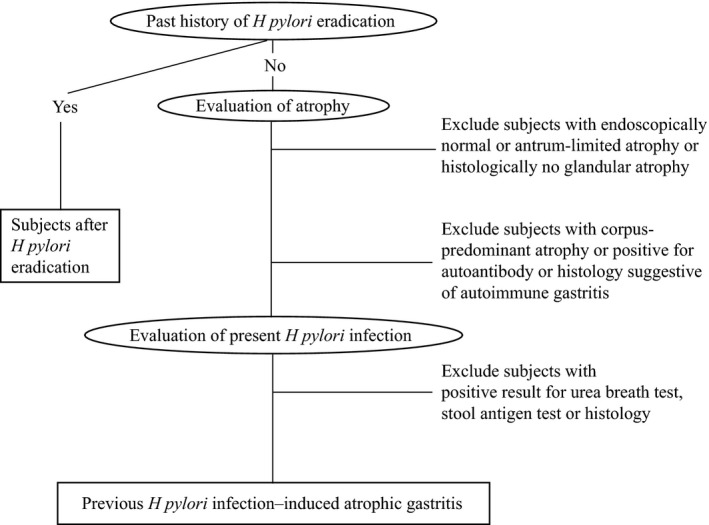
Flowchart to diagnose previous *H. pylori* infection–induced atrophic gastritis cases

A flowchart for the diagnosis is shown in Figure 1.

### 2.1 Evaluation of previous *H. pylori* eradication

Obtaining the patient's medical history of eradication is the first step in the diagnosis of unintended eradication. Clinicians must obtain a careful history regarding *H. pylori* eradication, including, for example, whether the patient had ever undergone eradication treatment, the treatment period, and whether the eradication treatment was successful or failed. Patients with a history of eradication treatment should not be considered to have previous *H. pylori* infection–induced atrophic gastritis.

### 2.2 Evaluation of chronic atrophic gastritis

The definition of chronic atrophic gastritis is not usually based on macroscopic findings; instead, it is based on histologic findings.^19^, ^20^ The most well‐known histologic criterion is the Sydney classification; however, other histologic staging systems (eg, Operative Link on Gastritis Assessment [OLGA] and Operative Link on Gastric intestinal metaplasia [OLGIM]) are also used for risk stratification.^20^, ^21^ Nordenstedt et al defined gastritis as “at least grade 1 neutrophils or mononuclear cells in the Sydney system in at least two gastric sites or at least grade 2 in at least one gastric site.”^22^

Therefore, we suggest that glandular atrophy of at least grade 1 in at least two gastric sites or at least grade 2 in at least one gastric site is the criterion for histologic atrophy, similar to the recommendations of Hiyama et al^14^ The recent increase in the use of antiplatelet or anticoagulant therapy^23^ makes it difficult to perform a biopsy merely for the evaluation of atrophy in all cases of endoscopy; therefore, it is not common. Several recent studies have suggested diagnostic concordance between endoscopic atrophy and histology.^24^, ^25^, ^26^

An atrophic border more severe than C2 in the Kimura‐Takemoto classification (atrophy limited to the gastric angle of the lower body) should be the minimum criterion for atrophic change when diagnosing unintended eradication based on the Kyoto classification.^20^, ^27^, ^28^ Recently, several investigators have reported that endoscopic staging using high‐resolution white light endoscopy plus virtual chromoendoscopy (Narrow Band Imaging, etc) is more accurate than white light endoscopy.^29^, ^30^, ^31^ Although the endoscopic diagnosis of atrophy may be feasible at experienced centers, it is difficult at centers that are less experienced with diagnosing atrophic gastritis endoscopically. Therefore, if an endoscopic diagnosis including chromoendoscopy is not possible, then histologically detected chronic atrophic gastritis is an option (Table 1).

The exclusion of autoimmune gastritis (0.49%‐1.1% in the general population) is another important step in the diagnosis of previous *H. pylori* infection–induced atrophic gastritis, because most subjects with autoimmune gastritis fulfill three out of four of our criteria.^32^, ^33^, ^34^ The endoscopic findings of corpus‐dominant atrophy with preservation of the antrum are characteristic of autoimmune gastritis and are diagnostic in clinical practice.^32^ The strict diagnosis of autoimmune gastritis should meet at least two criteria: positive specific autoantibodies to parietal cells or intrinsic factor and/or characteristic pathological features such as profound loss of oxyntic mucosa, infiltrates of lymphocytes and plasma cells in lamina propria, and enterochromaffin‐like (ECL) cell hyperplasia.^35^, ^36^, ^37^ However, it is not practical to evaluate autoantibodies and histology in daily clinical practice. We propose that exclusion of patients with suspected autoimmune gastritis with endoscopic findings is a minimum requirement, although this is practically difficult in some cases. Therefore, serology or histology to determine autoimmune gastritis is also desirable, especially for patients with severe atrophy who are negative for *H. pylori* infection (Table 1).

### 2.3 Negative diagnosis for present *H. pylori* infection

Strict exclusion of individuals with a present *H. pylori* infection is necessary, and we consider this the third step in the diagnosis. The widely recommended method to evaluate *H. pylori* status in patients post‐treatment is the 13C‐urea breath test (UBT), but the monoclonal stool antigen test (SAT) can be used alternatively.^38^ Previous investigators have reported high false‐negative UBT and SAT rates for patients using PPIs.^39^, ^40^ Therefore, PPIs should be discontinued for 2 weeks. Antibiotics and bismuth compounds should be stopped for at least 4 weeks to allow the detectable bacterial load to increase. Although serology is used for screening in clinical practice, and the results are not affected by medication, it cannot distinguish between present and previous infection because an antibody titer often remains positive even after successful eradication. Therefore, serology alone is not suitable for the evaluation of unintentional elimination. However, positive serology with negative results for another specific *H. pylori*‐detecting test (UBT or SAT) strongly suggests previous infection and elimination of *H. pylori* thereafter. If two diagnostic methods are available for use, then serology plus either UBT or SAT should be strongly considered (Table 1).

## 3 CHARACTERISTICS OF PATIENTS DEFINED AS HAVING PREVIOUS *H. pylori* INFECTION–INDUCED ATROPHIC GASTRITIS

Three different populations have been defined as having previous *H. pylori* infection–induced atrophic gastritis. They are described here.

### 3.1 Unintentional eradication without a history of eradication

Unintentionally *H. pylori*‐eradicated subjects without a history of eradication treatment comprise the first population; the majority of these subjects have chronic atrophic gastritis without a present *H. pylori* infection. Unintended *H. pylori* eradication could occur after exposure to antibiotics for another infectious disease. Low intragastric acidity is closely associated with the success of *H. pylori* eradication.^41^, ^42^ Furthermore, low intragastric acidity is induced by PPIs, and if antibiotics were incidentally administered to PPI‐treated subjects, then successful eradication may occur. However, standard PPI therapy often fails to maintain a long‐term increase in intragastric pH > 4.0, which is the minimum required environment for *H. pylori* eradication.^43^ Therefore, when subjects with severe gastric mucosal atrophy are treated with PPIs, unintended eradication may occur if they are incidentally administered antibiotics for other infectious diseases. Recently, the potassium‐competitive acid blocker vonoprazan has been used for acid‐related disease and *H. pylori* eradication in Japan. Vonoprazan provides more rapid and sustained inhibition of gastric acid secretion that is superior to that of PPIs.^44^ Therefore, eradication occurs in many subjects administered antibiotic treatment under acid inhibition by vonoprazan alone.

### 3.2 Unreported successful eradication

Subjects who fail to report *H. pylori* eradication despite a history of successful eradication treatment comprise the second population. This may occur due to an insufficient explanation of the eradication treatment from their physician, or the patient may have simply forgotten receiving eradication treatment. Unintentionally eradicated subjects (Section 3.1) and unreported successful eradication cases (Section 3.2) have the same pathophysiologic states because they were both eradicated by previous antibiotic use.

### 3.3 Spontaneous disappearance of * H. pylori*

The third population of subjects includes those who have experienced spontaneous disappearance of *H. pylori* due to the progression of atrophic gastritis.^45^, ^46^ Under physiological conditions, *H. pylori* survives in gastric epithelial cells; therefore, the loss of gastric epithelial cells induced by *H. pylori* itself leads to spontaneous elimination of *H. pylori*.^47^ This subgroup shows severely progressed atrophy and is similar to group D characterized by the ABC method (serologically atrophic PG and seronegative for *H. pylori*).^48^ Because the disappearance of *H. pylori* occurs independently of antibiotic use, the clinical background of this subgroup is quite different from both true unintended eradication cases and unreported successfully eradicated cases. However, it is possible that antibiotics were administered incidentally to patients with severely progressed atrophy under conditions of achlorhydria, resulting in eradication. The frequency of these subjects is low even in regions with a high *H. pylori* prevalence like East Asia.^49^, ^50^, ^51^, ^52^

It is important to note that these three types of populations cannot be distinguished from one another, even using endoscopy, serology, or a medical examination. There is a great difference in the prevalence of severe atrophy cases, but this is not a differential point in the diagnosis. We must not misunderstand that these cases are different categories of disease entities. In these cases, atrophic gastritis was induced by previous *H. pylori* infection, but living *H. pylori* do not exist, and the risk stratification should be based on the atrophic grade. Although most cases of spontaneous *H. pylori* disappearance (group D in the ABC classification) show severe atrophy,^45^, ^53^ the grades of atrophy of unintentionally eradicated cases and unreported eradication cases (group A in the ABC classification) depend on the atrophic status at the time of antibiotic administration, which varies for each case. Previous data indicated that the prevalence of severe atrophy after unintentional eradication among group A varies from 0% (0/20) reported by Chinda^17^ to 55.9% (19/34) analyzed by our previous study (H. Kishikawa, unpublished data),^54^ suggesting that the atrophic grade is milder than that of group D. Spontaneously disappeared cases tend to involve atrophic PG (PG I ≤ 70 ng/mL and PG I/II ratio ≤ 3.0), and unintended eradication tends to involve normal PG, which may also become the serologic differential point.

## 4 DIFFERENCE BETWEEN PREVIOUS *H. pylori* INFECTION–INDUCED ATROPHIC GASTRITIS AND *H. pylori*‐NEGATIVE GASTRITIS DEFINED IN WESTERN COUNTRIES

*Helicobacter pylori*‐negative gastritis is a recently defined disease entity diagnosed primarily based on histology in Western countries, which is similar but not identical to our criteria.^22^, ^55^, ^56^ The minimum required criterion of this disease entity is that *H. pylori* is not detected in the gastric mucosa despite typical histologic findings of chronic gastritis consistent with *H. pylori* infection, although some investigators define it using more strict criteria, including culture and serology. Although a major cause of *H. pylori*‐negative gastritis is unintended eradication, as suggested by Genta and Sonnenberg,^55^ a false‐negative *H. pylori* diagnosis caused by the suppression of *H. pylori* microorganisms in the gastric mucosa by PPI treatment or the unrelated use of antibiotics has also been regarded as a cause of *H. pylori*‐negative gastritis. Therefore, patients with a current *H. pylori* infection may be misclassified. Although PPI users are included in these studies, unintended elimination may represent an important cause of *H. pylori*‐negative gastritis; therefore, we included it in the list as a characteristics of previous *H. pylori* infection–induced atrophic gastritis (Table 2).

**Table 2:** Clinical characteristics of subjects with previous *H. pylori* infection–induced atrophic gastritis cases

Study	Source	N	Prevalence in the total population	Prevalence among the gastritis subjects	Evaluation of *H. pylori*	Evaluation of atrophy or gastritis	PPI subjects
Hiyama et al^14^	Consecutive outpatients in Vietnam	200	22/200 (11%)	22/142 (15.5%)	RUT, urine *H. pylori* antibody, and histology	RUT, urine *H. pylori* antibody, and histology	Excluded
Ono et al^15^	Patients with early gastric cancer treated by endoscopy in Japan	240	33/240 (13.8%)	n.a	RUT, histology, culture, and UBT	Histology, endoscopy and serology	Excluded
Boda et al^16^	Patients with early gastric cancer treated by endoscopy in Japan	270	27/271 (10%)	n.a	RUT, serology, histology, and UBT	Endoscopy and histology	Excluded
Nordenstedt et al^22^	Consecutive outpatients in USA	491	41/491 (8.4%)	41/200 (20.5%)	Histology, culture, and serology	Histology	Not excluded
Genta et al^55^	National pathology database in USA	895323	13829/895323 (1.5%)	13829/108833 (12.7%)	Histology	Histology	Not excluded
Shiota et al^56^	Consecutive outpatients in USA	1240	123/1240 (9.9%)	123/695 (17.7%)	Histology, culture, and serology	Histology	Not excluded

Abbreviations: RUT, rapid urease test; UBT, urea breath test; PPI, proton‐pump inhibitor.

## 5 EPIDEMIOLOGY OF PREVIOUS *H. pylori* INFECTION–INDUCED ATROPHIC GASTRITIS

Previously reported clinical characteristics of previous *H. pylori* infection–induced atrophic gastritis cases are shown in Table 2. Hiyama et al found that unintentionally eradicated subjects accounted for 11% (22/200) of consecutive patients without a history of *H. pylori* eradication in Vietnam; this is the only report concerning the prevalence of unintentional elimination in the general population that has excluded PPI users.^14^ In their report, 22 of 142 *H. pylori‐*related gastritis patients composed of present *H. pylori* infection (n = 120) and unintended *H. pylori* eradication (n = 22) were considered unintentionally eliminated cases. Recently, Kaji et al analyzed negative *H. pylori* infection cases and reported that endoscopically atrophic cases (more than C2) among negative *H. pylori* infection cases comprised 8.4% of all atrophic gastritis cases (602/7201), which was a lower percentage than that of Hiyama et al^57^ However, because *H. pylori* infection was evaluated merely by an examination, the results may be regarded as unconfirmed.^57^ Other studies performed in Japan reported an unexpectedly high prevalence of *H. pylori*‐negative subjects among gastric cancer patients. A report by Matsuo et al indicated that the prevalence of true *H. pylori*‐negative gastric cancer is extremely low, approximately 0.66% in Japan. This suggested that almost all patients with gastric cancer in Japan are likely to have a current *H. pylori* infection or previously had one.^58^ However, Ono et al reported that 33 of 240 early gastric cancer patients (12.2%) showed histologic atrophy and intestinal metaplasia despite no current *H. pylori* infection.^15^ Boda et al reported a similar result with 27 of 271 patients with early stage gastric cancer (approximately 10%) showing endoscopic atrophy and histologic atrophic changes despite negative *H. pylori* serology and histology.^16^ These reports suggested that approximately 10%‐12% of early gastric cancer patients in Japan are unintentionally eliminated cases.

In Western countries where *H. pylori* infection rates are lower than in East Asia, the prevalence of “*H. pylori* negative gastritis” has been described by several investigators. Shiota et al reported that 17.7% of all patients with gastritis had *H. pylori*‐negative gastritis.^56^ Similar rates were reported by Nordenstedt (20.5%)^22^ and Genta (12.7%).^55^ These reports were based on populations in Western countries. The prevalence of reported eliminated *H. pylori* cases in East Asia is 15.5% of *H. pylori‐*related gastritis cases (22/142),^14^ which was similar to that of Western countries, suggesting that approximately 10%‐20% of gastritis cases are unintentionally eliminated cases in all regions irrespective of the *H. pylori* infection rate.

## 6 REPORTED RATE OF UNINTENTIONAL ELIMINATION

Several investigators have reported the annual unintentional elimination rate of *H. pylori* infection in adults, often using the term “seroreversion rate” (Table 3). However, previous *H. pylori* infection–induced atrophic gastritis cases are a broader entity because the *H. pylori* antibody does not seroconvert in all eradicated subjects. Kikuchi et al reported that seroreversion rates over a 9‐year period were 6.3%, with rates of 7.9 per 1000 person‐years (95% confidence interval: 5.2‐8.7) for Japanese workers undergoing serologic evaluation.^59^ Jung et al performed a retrospective cohort study of healthy adults in Korea and found an annual seroconversion rate of 2.42%.^60^ To date, a wide range of seroreversion rates have been reported by several other investigators, such as 1.5% per year,^61^ 7.7% in 11 years,^62^ 0.11%‐0.35% per person‐year,^63^ and 1 per 100 person‐years.^64^ Considering the aforementioned annual seroreversion rate of approximately 1%‐3%, the unintentional elimination rate of approximately 10% seems disproportionately high and should be investigated further.

**Table 3:** Reported unintended elimination (seroreversion) rates in adults

Study	Source	N	Mean observation period	Unintended eradication rate	Evaluation of *H. pylori*
Kikuchi et al^59^	Workers visiting for health check‐up	1286	9 years	7.9 per 1000 person‐years	Serology
Jung et al^60^	Healthy subjects visiting health screening center	67 212	4.6 years	2.42% per 1 year	Serology
Kumagai et al^61^	644 children and adults in Japan	644	8 years	1.5% per 1 year	Serology
Rosenstock et al^62^	Random sample of Danish subjects	529	11 years	7.7% in 11 years	Serology
Fawcett et al^63^	Subjects born in 1972‐3	452	5 years	0.11%‐0.35% per person‐year	Serology
Bastos et al^64^	Noninstitutionalized adults	2067	3 years	1 per 100 person‐years	Serology

## 7 SEROLOGIC CHARACTERISTICS OF PATIENTS WITH PREVIOUS *H. pylori* INFECTION–INDUCED ATROPHIC GASTRITIS CASES

### 7.1 Serologic characteristics of unintended or unreported eradication

The ABC method is used to screen for serum gastric cancer with anti‐*H. pylori* serology in the form of anti‐*H. pylori* IgG antibody titers and atrophic gastritis detected by serum PG. Subjects are classified into four groups: group A [*H. pylori* (−)PG(−)], *H. pylori* infection‐free healthy stomachs; group B [*H. pylori* (+)PG(−)], *H. pylori*‐infected subjects without extensive chronic atrophic gastritis (CAG); group C [*H. pylori* (+)PG(+)], *H. pylori*‐induced extensive CAG; and group D [*H. pylori* (−)PG(+)], subjects with spontaneous disappearance of *H. pylori* antibody titer and severe CAG with extensive intestinal metaplasia.^65^, ^66^, ^67^

With the ABC method, most previous *H. pylori* infection–induced atrophic gastritis cases are classified as “normal” (group A) or as the high‐risk group (group D). Based on our previous report, 71% of *H. pylori*‐positive subjects were classified as group A after successful eradication within 2 years.^54^

Several studies have reported a cutoff value to distinguish unintended eradication cases among group A subjects, which is defined as normal PG and seronegative for *H. pylori*. First, we reported that PGI levels ≤ 37 ng/mL and PGI/II ratios ≤ 5.1 effectively identified unintentionally eradicated cases in group A.^54^ We also suggested that a PGI/II ratio ≤ 4.3 and *H. pylori* antibody titer ≥ 3.0 were independent predictor of gastric neoplasia in patients serologically classified as group A,^68^ and all of these cases showed atrophy on endoscopy, suggesting that they indeed were unintentionally eradicated cases. Chinda et al also reported similar cutoff values of PGI and the PGI/II ratio for determining unintentionally eradicated cases.^17^ The cutoff values of PGI and the PGI/II ratio in this study were ≤ 31.2 ng/mL and ≤ 4.6, respectively.

Kikuchi et al showed that a PGII value ≤ 10 ng/mL or PGI/II ratio ≤ 5.0 is the optimal criterion for differentiating never‐infected versus infected and formerly infected subjects. 69 Kitamura et al demonstrated that *H. pylori* infection status can be differentiated by a PGI/II ratio ≤ 4.5, with sensitivity and specificity values > 80%.^70^ (Table 4).

**Table 4:** Criteria of serum pepsinogens to discriminate previous *H. pylori* infection–induced atrophic gastritis cases

Study	Comparison target		Results
Chinda et al^17^	*H. pylori ‐*uninfected cases	Unintentionally *H. pylori‐*eliminated cases	PGI ≤ 31.2 ng/ml, PGI/II ratio ≤ 4.6
Kishikawa et al^68^		Successfully *H. pylori‐*eradicated cases	PGI ≤ 37 ng/ml, PGI/II ratio ≤ 5.1
Kikuchi et al^69^		Both *H. pylori*‐infected and formerly infected cases	PGII ≥ 10 ng/ml, PGI/II ratio ≤ 5
Kitamura et al^70^		Both *H. pylori*‐infected and formerly infected cases	PGI/II ratio ≤ 4.5
Miki et al^67^		Spontaneously resolved *H. pylori* cases	PGI ≤ 70 ng/ml, PGI/II ratio ≤ 3

Abbreviation: PG, pepsinogen.

### 7.2 Serologic characteristics of spontaneously disappeared cases

With the ABC classification, PGI ≤ 70 ng/mL and PGI/II ratio ≤ 3 with negative *H. pylori* serology are the classical criteria of group D, defined as unintentional disappearance due to severe atrophy (Table 4).^67^In our preliminary evaluation, the prevalence rate of autoimmune gastritis was approximately 30% in group D (data not shown). Given the difficulty of endoscopically diagnosing autoimmune gastritis and the high prevalence in group D, measuring anti‐parietal cell antibody levels is useful especially for cases of severe atrophy and negative *H. pylori* serology, including group D, as discussed in Section 2.2.

Several cutoff values (PGI ≤ 31‐37 ng/mL or PGI/II ratio ≤ 4.3‐5.1; or PGII value ≤ 10 ng/mL) can be used to distinguish unintentionally eradicated cases in group A. These cutoff values are applicable for differentiating unintentionally eradicated cases from serologically normal subjects. PGI ≤ 70 ng/mL, PGI/II ratio ≤ 3, and negative *H. pylori* serology are also regarded as cutoff values for spontaneously disappeared cases (group D) (Table 4). It should be noted that these cutoff values have only a subsidiary role in the diagnosis of unintentional elimination in clinical practice.

## 8 CHARACTERISTICS OF GASTRIC CANCER IN PREVIOUS *H. pylori* INFECTION–INDUCED ATROPHIC GASTRITIS

Endoscopic characteristics of gastric cancer after eradication, which are similar to those of unintentionally eradicated cases of *H. pylori*, have been discussed by several investigators. Difficulty diagnosing cancer itself has been reported due to non‐neoplastic epithelium histologically appearing on the lesion surface after eradication.^71^

The most reported histologic feature of gastric cancer in cases after successful eradication is differentiated type (75%; 15/20).^72^ The rates of differentiated type cancer in gastric cancer cases classified as group A range from 86.9% (93/104)^18^ to 88.9% (8/9),^68^ and that of group D subjects has been reported as 83.3% (10/12).^73^ The characteristics of gastric cancer in cases of previous *H. pylori* infection–induced atrophic gastritis include difficult visual recognition by endoscopy and histologically differential type.

## 9 RISK OF GASTRIC CANCER DEVELOPMENT IN PREVIOUS *H. pylori* INFECTION–INDUCED ATROPHIC GASTRITIS AND INTERVALS OF ENDOSCOPIC SURVEILLANCE

The risk of gastric cancer in unintentionally eradicated subjects is theoretically equal to that of successfully eradicated cases. However, there is no established interval or risk stratification method for endoscopy.^74^ The effectiveness of *H. pylori* eradication for the prevention of gastric cancer depends on the severity of atrophy at the time of eradication.^75^, ^76^ Only two reports have indicated the development rate of gastric cancer in non‐cancer severe atrophic gastritis cases after successful eradication; these were 0.31%^57^ to 0.62%^75^ per year.

The risk of gastric cancer development in spontaneously disappeared cases (group D) has been evaluated by several investigators; these rates have been reported as 0.53%,^77^ 0.60%,^73^ 0.67%,^51^ and 0.87%^78^ per year. These reports suggested that patients with severe atrophy are at high risk of gastric carcinogenesis, as approximately 10%‐20% of individuals develop cancer during the 30 years after eradication; therefore, endoscopic surveillance is justified. Studies have also suggested that individuals with mild or no atrophy are at low risk and that endoscopic surveillance is not justified. The risk of gastric cancer development in post‐eradicated cases with severe atrophy (0.31%‐0.62% per year^57^, ^75^) is slightly lower than that of group D patients (0.53%‐0.87% per year^51^, ^73^, ^77^, ^78^) which is compatible with the prevalence of severe atrophy cases. It should be noted that the atrophy grade is the key factor when stratifying future gastric development risk.

Intestinal metaplasia is another established finding that can predict gastric cancer development, and the utility of endoscopic diagnosis of intestinal metaplasia, especially using chromoendoscopy, has been recognized.^79^

Recently, Cheung et al reported that long‐term use of PPIs was associated with gastric cancer risk, even after *H. pylori* eradication, during a median follow‐up of 7.6 years (hazard ratio: 2.44).^80^, ^81^, ^82^ However, performing regular endoscopic surveillance of PPI users may be excessive. Therefore, chronic atrophic gastritis and intestinal metaplasia are considered high‐risk criteria for gastric cancer. Based on recent guidelines, we recommend endoscopy every 3 years for patients with severe atrophy (>O1 according to the Kimura‐Takemoto classification; atrophic border does not cross the lesser curvature of the stomach but extends along the anterior or posterior of the stomach), or for those with endoscopically or histologically detected intestinal metaplasia.^83^, ^84^ Endoscopic surveillance of high‐risk patients with unintentionally eliminated cases is effective in East Asia, where more than half of all gastritis cases show advanced atrophy.^85^ However, we consider this strategy useful even in Western countries, where approximately 20% of gastritis patients exhibit advanced atrophy,^86^ because of the extremely high rate of gastric cancer development in high‐risk cases, and the difficulty diagnosing gastric cancer in patients after eradication.

## 10 CONCLUSION

Individuals with atrophic gastric mucosa but no current *H. pylori* infection and no history of eradication therapy have been identified. If false‐negative cases and autoimmune gastritis cases are excluded, then atrophic gastritis in these individuals is induced by previous *H. pylori* but no living *H. pylori* organisms exist. Herein, we defined previous *H. pylori* infection–induced atrophic gastritis as fulfilling the following conditions: no history of eradication; changes in atrophy confirmed by endoscopy or histology; negative for active *H. pylori* infection; and absence of autoimmune gastritis diagnosed by endoscopy, autoantibodies, or characteristic histology. Approximately 10% of early gastric cancer cases resected by endoscopy are potentially unintended elimination cases in areas with a high prevalence of *H. pylori* infection. Approximately 10%‐20% of histologic gastritis cases are also regarded as unintentionally eliminated cases, irrespective of the *H. pylori* infection rate.

Three different populations are inevitably included among unintended eradication cases defined using the aforementioned criteria: individuals with unintentionally eradicated *H. pylori*, who comprise the majority; individuals who failed to report *H. pylori* eradication despite successful eradication treatment; and individuals who experience spontaneous disappearance of *H. pylori* due to the progression of atrophic gastritis. The prevalence of severe atrophy is significantly high in spontaneously disappeared case. These subgroups cannot be distinguished even with endoscopy, serology, or an examination; however, the PG test might be a diagnostic modality that can differentiate spontaneously disappeared cases.

When serologic gastric cancer screening was performed using PG and *H. pylori* serology (ABC method), most subjects with previous *H. pylori* infection–induced atrophic gastritis were classified as normal (group A) or as the high‐risk group (group D); therefore, several cutoff values to identify the unintended eradication cases have been proposed. PGI ≤ 31.2‐37 ng/mL, PGI/II ratio ≤ 4.3‐5.1, and PGII ≤ 10 ng/mL are the suggested cutoff values based on previous reports of misclassified subjects in group A, and PGI ≤ 70 ng/mL and PGI/II ratio ≤ 3 are the suggested cutoff values for subjects with spontaneous disappearance case classified as group D. Despite the significantly different prevalence of severe atrophy, this population should be the regarded as having a single disease entity because atrophy is induced by *H. pylori*, and the atrophic grade is especially important in risk stratification. Therefore, we recommend performing endoscopy every 3 years for higher‐risk patients with severe atrophy and intestinal metaplasia. However, surveillance endoscopy is not justified for low‐risk patients. Because gastric cancer in previous infection cases is difficult to diagnose endoscopically, careful endoscopic surveillance based on the guidelines may aid in the early detection of gastric cancer in this overlooked high‐risk patient population.

## CONFLICT OF INTEREST

The authors have no competing interests.

## References

[1] International Agency for Research on Cancer . Schistosomes, liver flukes and *Helicobacter pylori* . IARC Monogr Eval Carcinog Risks Hum. 1994;61:177‐241. 7715068 PMC7681621

[2] The EUROGAST Study Group . An international association between *Helicobacter pylori* infection and gastric cancer. Lancet. 1993;341:1359‐1362. 8098787

[3] Uemura N , Okamoto S , Yamamoto S , et al. *Helicobacter pylori* infection and the development of gastric cancer. N Eng J Med. 2001;345:784‐789. 10.1056/NEJMoa001999 11556297

[4] Choi IJ , Kook MC , Kim YI , et al. *Helicobacter pylori* therapy for the prevention of metachronous gastric cancer. N Eng J Med. 2018;378:1085‐1095. 10.1056/NEJMoa1708423 29562147

[5] Franco AT , Johnson E , Krishna U , et al. Regulation of gastric carcinogenesis by *Helicobacter pylori* virulence factors. Cancer Res. 2008;68:379‐387. 18199531 10.1158/0008-5472.CAN-07-0824 PMC3114460

[6] Correa P . Human gastric carcinogenesis: a multistep and multifactorial process–First American Cancer Society Award Lecture on Cancer Epidemiology and Prevention. Cancer Res. 1992;52:6735‐6740. 1458460

[7] Ford AC , Gurusamy KS , Delaney B , Forman D , Maoyyedi P . Eradication therapy for peptic ulcer disease in *Helicobacter pylori*‐positive people. Cochrane Database Syst Rev. 2016;4:CD003840. 27092708 10.1002/14651858.CD003840.pub5 PMC7163278

[8] Nakamura S , Sugiyama T , Matsumoto T , et al. Long‐term clinical outcome of gastric MALT lymphoma after eradication of *Helicobacter pylori*: a multicenter cohort follow‐up study of 420 patients in Japan. Gut. 2012;61:507‐513. 21890816 10.1136/gutjnl-2011-300495

[9] Leung WK , Lin S‐R , Ching JYL , et al. Factors predicting progression of gastric intestinal metaplasia: Results of a randomised trial on *Helicobacter pylori* eradication. Gut. 2004;53:1244‐1249. 15306578 10.1136/gut.2003.034629 PMC1774213

[10] Ford AC , Forman D , Richard H , Yuan Y , Moayyedi P *Helicobacter pylori* eradication therapy to prevent gastric cancer in healthy asymptomatic infected individuals: systematic review and meta‐analysis of randomized controlled trials. BMJ. 2014;348:g3174. 24846275 10.1136/bmj.g3174 PMC4027797

[11] Fukase K , Kato M , Kikuchi S , et al. Effect of eradication of *Helicobacter pylori* on incidence of metachronous gastric carcinoma after endoscopic resection of early gastric cancer: an open‐label, randomized controlled trial. Lancet. 2008;372:392‐397. 18675689 10.1016/S0140-6736(08)61159-9

[12] Yoon SB , Park JM , Lim CH , Cho YK , Choi MG . Effect of *Helicobacter pylori* eradication on metachronous gastric cancer after endoscopic resection of gastric tumors: a meta‐analysis. Helicobacter. 2014;19:243‐248. 25056262 10.1111/hel.12146

[13] Herroro R , Park JY , Forman D . The fight against gastric cancer ‐ the IARC working group report. Best Pract Res Clin Gastroenterol. 2014;28:1107‐1114. 25439075 10.1016/j.bpg.2014.10.003

[14] Hiyama T , Quach DT , Le QD , et al. Rate of unintended *Helicobacter pylori* eradication in the Vietnamese. Helicobacter. 2015;20:156‐157. 25660825 10.1111/hel.12210

[15] Ono S , Kato M , Suzuki M , et al. Frequency of *Helicobacter pylori*‐negative gastric cancer and gastric mucosal atrophy in a Japanese endoscopic submucosal dissection series including histological, endoscopic and serological atrophy. Digestion. 2012;86:59‐65. 22722747 10.1159/000339176

[16] Boda T , Ito M , Yoshihara M , et al. Advanced method for evaluating of gastric cancer risk by serum marker: determination of true low‐risk subjects for gastric neoplasia. Helicobacter. 2013;19:1‐8. 24215601 10.1111/hel.12101

[17] Chinda D , Shimoyama T , Mikami T , et al. Serum pepsinogen levels indicate the requirement of upper gastrointestinal endoscopy among Group A subjects of ABC classification: a multicenter study. J Gastroenterol. 2018;53:924‐931. 29353347 10.1007/s00535-018-1431-9

[18] Kiso M , Yoshihara M , Ito M , et al. Characteristics of gastric cancer in negative test of serum anti‐*Helicobacter pylori* antibody and pepsinogen‐test: a multicenter study. Gastric Cancer. 2017;20:764‐771. 28025702 10.1007/s10120-016-0682-5

[19] Rugge M , Correa P , Dixon MF , et al. Gastric mucosal atrophy: interobserver consistency using new criteria for classification and grading. Aliment Pharmacol Ther. 2002;16:1249‐1259. 12144574 10.1046/j.1365-2036.2002.01301.x

[20] Sugano K , Tack J , Kuipers EJ , et al. Kyoto global consensus report on *Helicobacter pylori* gastritis. Gut. 2015;64:1353‐1367. 26187502 10.1136/gutjnl-2015-309252 PMC4552923

[21] Dixon MF , Genta RM , Yardley JH , Correa P . Classification and grading of gastritis. The updated Sydney System. International Workshop on the Histopathology of Gastritis, Houston 1994. Am J Surg Pathol. 1996;20:1161‐1181. 8827022 10.1097/00000478-199610000-00001

[22] Nordenstedt H , Graham DY , Kramer JR , et al. *Helicobacter pylori*‐negative gastritis: prevalence and risk factors. Am J Gastroenterol. 2013;108:65‐71. 23147524 10.1038/ajg.2012.372 PMC3984401

[23] Williams CD , Chan AT , Elman MR , et al. Aspirin use among adults in the U.S.: results of a national survey. Am J Prev Med. 2015;48:501‐508. 25891049 10.1016/j.amepre.2014.11.005

[24] Kono S , Gotoda T , Yoshida S , et al. Can endoscopic atrophy predict histological atrophy? Historical study in United Kingdom and Japan. World J Gastroenterol. 2015;21:13113‐13123. 26673849 10.3748/wjg.v21.i46.13113 PMC4674730

[25] Liu Y , Uemura N , Xiao SD , Tytgat GN , Kate FJ . Agreement between endoscopic and histological gastric atrophy scores. J Gastroenterol. 2005;40:123‐127. 15770394 10.1007/s00535-004-1511-x

[26] Mihara M , Haruma K , Kamada T , et al. The role of endoscopic findings for the diagnosis of *Helicobacter pylori* infection: evaluation in a country with high prevalence of atrophic gastritis. Helicobacter. 1999;4:40‐48. 10352086 10.1046/j.1523-5378.1999.09016.x

[27] Kimura K , Takemoto T . An endoscopic recognition of the atrophic border and its significance in chronic gastritis. Endoscopy. 1969;3:87‐97.

[28] Sugimoto M , Ban H , Ichikawa H , et al. Efficacy of the Kyoto classification of gastritis in identifying patients at high risk for gastric cancer. Intern Med. 2017;56:579‐586. 28321054 10.2169/internalmedicine.56.7775 PMC5410464

[29] Pimentel‐Nunes P , Libanio D , Lage J , et al. A multicenter prospective study of the real‐time use of narrow‐band imaging in the diagnosis of premalignant gastric conditions and lesions. Endoscopy. 2016;48:723‐730. 27280384 10.1055/s-0042-108435

[30] Saka A , Yagi K , Nimura S . OLGA‐ and OLGIM‐based staging of gastritis using narrow‐band imaging magnifying endoscopy. Dig Endosc. 2015;27:734‐741. 25923666 10.1111/den.12483

[31] East JE , Vleugels JL , Roelandt P , et al. Advanced endoscopic imaging: European society of gastrointestinal endoscopy (ESGE) technology review. Endoscopy. 2016;48:1029‐1045. 27711949 10.1055/s-0042-118087

[32] Neumann WL , Coss E , Rugge M , Genta RM . Autoimmune atrophic gastritis–pathogenesis, pathology and management. Nat Rev Gastroenterol Hepatol. 2013;10:529‐541. 23774773 10.1038/nrgastro.2013.101

[33] Notsu T , Adachi K , Mishiro T , et al. Prevalence of autoimmune gastritis in individuals undergoing medical checkups in Japan. Intern Med. 2019;58:1817‐1823. 30918182 10.2169/internalmedicine.2292-18 PMC6663548

[34] Park JY , Cornish TC , Lam‐Himlin D , Shi C , Montgomery E . Gastric lesions in patients with autoimmune metaplastic atrophic gastritis (AMAG) in a tertiary care setting. Am J Surg Pathol. 2010;34:1591‐1598. 20975338 10.1097/PAS.0b013e3181f623af

[35] Soykan I , Yakut M , Keskin O , Bektas M . Clinical profiles, endoscopic and laboratory features and associated factors in patients with autoimmune gastritis. Digestion. 2012;86:20‐26. 22710370 10.1159/000338295

[36] Antico A , Tampoia M , Villalta D , Tonutti E , Tozzoli R , Bizzaro N . Clinical usefulness of the serological gastric biopsy for the diagnosis of chronic autoimmune gastritis. Clin Dev Immunol. 2012;2012:520970. 23251219 10.1155/2012/520970 PMC3520153

[37] Rugge M , Fassan M , Pizzi M , et al. Autoimmune gastritis: histology phenotype and OLGA staging. Aliment Pharmacol Ther. 2012;35:1460‐1466. 22519568 10.1111/j.1365-2036.2012.05101.x

[38] Malfertheiner P , Megraud F , O’Morain CA , et al. Management of *Helicobacter pylori* infection‐the Masstricht V/Florence Consensus report. Gut. 2017;66:6‐30. 27707777 10.1136/gutjnl-2016-312288

[39] Levine A , Shevah O , Shabat‐Sehayek V , et al. Masking of 13C urea breath test by proton pump inhibitors is dependent on type of medication: comparison between omeprazole, pantoprazole, lansoprazole and esomeprazole. Aliment Pharmacol Ther. 2004;20:117‐122. 15225178 10.1111/j.1365-2036.2004.02021.x

[40] Gatta L , Vakil N , Ricci C , et al. Effect of proton pump inhibitors and antacid therapy on 13C urea breath tests and stool test for *Helicobacter pylori* infection. Am J Gastroenterol. 2004;99:823‐829. 15128344 10.1111/j.1572-0241.2004.30162.x

[41] Yang JC , Wang HL , Chern HD , et al. Role of omeprazole dosage and cytochrome P450 2C19 genotype in patients receiving omeprazole‐amoxicillin dual therapy for *Helicobacter pylori* eradication. Pharmacotherapy. 2011;31:227‐238. 21361732 10.1592/phco.31.3.227

[42] Sugimoto M , Shirai N , Nishino M , et al. Rabeprazole 10 mg q.d.s. decreases 24‐h intragastric acidity significantly more than rabeprazole 20 mg b.d. or 40 mg o.m., overcoming CYP2C19 genotype. Aliment Pharmacol Ther. 2012;36:627‐634. 22882464 10.1111/apt.12014

[43] Sahara S , Sugimoto M , Uotani T , et al. Twice‐daily dosing of esomeprazole effectively inhibits acid secretion in CYP2C19 rapid metabolisers compared with twice‐daily omeprazole, rabeprazole or lansoprazole. Aliment Pharmacol Ther. 2013;38:1129‐1137. 24099474 10.1111/apt.12492

[44] Kawai T , Oda K , Funao N , et al. Vonoprazan prevents low‐dose aspirin‐associated ulcer recurrence: randomised phase 3 study. Gut. 2018;67:1033‐1041. 29196436 10.1136/gutjnl-2017-314852 PMC5969345

[45] Kokkola A , Kosunen TU , Puolakkainen P , et al. Spontaneous disappearance of *Helicobacter pylori* antibodies in patients with advanced atrophic corpus gastritis. APMIS. 2003;111:619‐624. 12969017 10.1034/j.1600-0463.2003.1110604.x

[46] Xia HH , Talley NJ . Natural acquisition and spontaneous elimination of *Helicobacter pylori* infection: clinical implications. Am J Gastroenterol. 1997;92:1780‐1787. 9382036

[47] Rugge M , Di Mario F , Cassaro M , et al. Pathology of the gastric antrum and body associated with *Helicobacter pylori* infection in non‐ulcerous patients: is the bacterium a promoter of intestinal metaplasia? Histopathology. 1993;22:9‐15. 8436350 10.1111/j.1365-2559.1993.tb00062.x

[48] Kishikawa H , Kimura K , Takarabe S , Kaida S , Nishida J . *Helicobacter pylori* antibody titer and gastric cancer screening. Dis Markers. 2015;2015:156719. 26494936 10.1155/2015/156719 PMC4606161

[49] Mizuno S , Miki I , Ishida T , et al. Prescreening of a high‐risk group for gastric cancer by serologically determined *Helicobacter pylori* infection and atrophic gastritis. Dig Dis Sci. 2010;55:3132‐3137. 20204698 10.1007/s10620-010-1154-0

[50] Charvat T , Sasazuki S , Inoue M , et al. Prediction of the 10‐year probability of gastric cancer occurrence in the Japanese population: the JPHC study cohort II. Int J Cancer. 2016;138:320‐331. 26219435 10.1002/ijc.29705

[51] Ikeda F , Shikata K , Hata J , et al. Combination of *Helicobacter pylori* antibody and serum pepsinogen as a good predictive tool of gastric cancer incidence: 20‐year prospective data from the Hisayama study. J Epidemiol. 2016;26:629‐636. 27265836 10.2188/jea.JE20150258 PMC5121431

[52] Song M , Camargo MC , Weinstein SJ , et al. Serum pepsinogen 1 and anti‐*Helicobacter pylori* IgG antibodies as predictors of gastric cancer risk in Finnish males. Aliment Pharmacol Ther. 2018;47:494‐503. 29243850 10.1111/apt.14471 PMC5776724

[53] Kotachi T , Ito M , Yoshihara M , et al. Serological evaluation of gastric cancer risk based on pepsinogen and *Helicobacter pylori* antibody: relationship to endoscopic findings. Digestion. 2017;95:314‐318. 28571035 10.1159/000477239

[54] Kishikawa H , Kimura K , Ito A , et al. Cutoff pepsinogen level for predicting unintendedly eradicated cases of *Helicobacter pylori* infection in subjects with seemingly normal pepsinogen levels. Digestion. 2017;95:229‐236. 28355604 10.1159/000469705

[55] Genta RM , Sonnenberg A . Helicobacter‐negative gastritis: a distinct entity unrelated to *Helicobacter pylori* infection. Aliment Pharmacol Ther. 2015;41:218‐226. 25376264 10.1111/apt.13007

[56] Shiota S , Thrift AP , Green L , et al. Clinical manifestations of *Helicobacter pylori*‐negative gastritis. Clin Gastroenterol Hepatol. 2017;15:1037‐1046. 28110098 10.1016/j.cgh.2017.01.006

[57] Kaji K , Hashiba A , Uotani C , et al. Grading of atrophic gastritis is useful for risk stratification in endoscopic screening for gastric cancer. Am J Gastroenterol. 2019;114:71‐79. 30315306 10.1038/s41395-018-0259-5

[58] Matsuo T , Ito M , Takata S , Tanaka S , Yoshihara M , Chayama K . Low prevalence of *Helicobacter pylori*‐negative gastric cancer among Japanese. Helicobacter. 2011;16:415‐419. 22059391 10.1111/j.1523-5378.2011.00889.x

[59] Kikuchi S , Ohgihara A , Hasegawa A , Miki K , Kaneko E , Mizokoshi H . Seroconversion and seroreversion of *Helicobacter pylori* antibodies over a 9‐year period and related factors in Japanese adults. Helicobacter. 2004;9:335‐341. 15270748 10.1111/j.1083-4389.2004.00233.x

[60] Jung JH , Choi KD , Han S , et al. Seroconversion rates of *Helicobacter pylori* infection in Korean adults. Helicobacter. 2013;18:299‐308. 23521610 10.1111/hel.12043

[61] Kumagai T , Malaty HM , Graham DY , et al. Acquisition versus loss of *Helicobacter pylori* infection in Japan: results from an 8‐year birth cohort study. J Infect Dis. 1998;178:717‐721. 9728540 10.1086/515376

[62] Rosenstock S , Jorgensen T , Andersen L , Bonnevie O . Seroconversion and seroreversion in IgG antibodies to *Helicobacter pylori*: a serology based prospective cohort study. J Epidemiol Community Health. 2000;54:444‐450. 10818120 10.1136/jech.54.6.444 PMC1731697

[63] Fawcett JP , Barbezat GO , Poulton R , Milne BJ , Xia HH , Talley NJ . *Helicobacter pylori* serology in a birth cohort of New Zealanders from age 11 to 26. World J Gastroenterol. 2005;11:3273‐3276. 15929181 10.3748/wjg.v11.i21.3273 PMC4316062

[64] Bastos J , Peleterio B , Barros R , et al. Sociodemographic determinants of prevalence and incidence of *Helicobacter pylori* infection in Portuguese adults. Helicobacter. 2013;18:413‐422. 23725608 10.1111/hel.12061

[65] Yamaguchi Y , Nagata Y , Hiratsuka R , et al. Gastric cancer screening by combined assay for serum anti‐*Helicobacter pylori* IgG antibody and serum pepsinogen levels‐the ABC method. Digestion. 2016;93:13‐18. 26789514 10.1159/000441742

[66] Yoshida T , Kato J , Inoue I , et al. Cancer development based on chronic active gastritis and resulting gastric atrophy as assessed by serum levels of pepsinogen and *Helicobacter pylori* antibody titer. Int J Cancer. 2014;134:1445‐1457. 24009139 10.1002/ijc.28470

[67] Miki K . Gastric cancer screening by combined assay for serum anti‐*Helicobacter pylori* IgG antibody and serum pepsinogen levels ‐ “ABC method”. Proc Jpn Acad Ser B Phys Biol Sci. 2011;87:405‐414. 10.2183/pjab.87.405 PMC3171284 21785258

[68] Kishikawa H , Kimura K , Ito A , et al. Predictors of gastric neoplasia in cases negative for *Helicobacter pylori* antibody and with normal pepsinogen. Anticancer Res. 2015;35:6765‐6771. 26637894

[69] Kikuchi S , Kato M , Mabe K , et al. Optimal criteria and diagnostic ability of serum pepsinogen values for *Helicobacter pylori* infection. J Epidemiol. 2019;29:147‐154. 30249942 10.2188/jea.JE20170094 PMC6414809

[70] Kitamura Y , Yoshihara M , Ito M , et al. Diagnosis of *Helicobacter pylori*‐induced gastritis by serum pepsinogen levels. J Gastrotenterol Hepatol. 2015;30:1473‐1477. 10.1111/jgh.12987 25974661

[71] Shichijo S , Hirata Y . Characteristics and predictors of gastric cancer after *Helicobacter pylori* eradication. World J Gastroenterol. 2018;24:2163‐2172. 29853734 10.3748/wjg.v24.i20.2163 PMC5974578

[72] Kamada T , Hata J , Sugiu K , et al. Clinical features of gastric cancer discovered after successful eradication of *Helicobacter pylori*: results from a 9‐year prospective follow‐up study in Japan. Aliment Pharmacol Ther. 2005;21:1121‐1126. 15854174 10.1111/j.1365-2036.2005.02459.x

[73] Watabe H , Mitsushima T , Yamaji Y , et al. Predicting the development of gastric cancer from combining *Helicobacter pylori* antibodies and serum pepsinogen status: a prospective endoscopic cohort study. Gut. 2005;54:764‐768. 15888780 10.1136/gut.2004.055400 PMC1774550

[74] Chen HN , Wang Z , Li X , Zhou ZG *Helicobacter pylori* eradication cannot reduce the risk of gastric cancer in patients with intestinal metaplasia and dysplasia: evidence from a meta‐analysis. Gastric Cancer. 2016;19:166‐175. 25609452 10.1007/s10120-015-0462-7

[75] Take S , Mizuno M , Ishiki K , et al. The long‐term risk of gastric cancer after the successful eradication of *Helicobacter pylori* . J Gastrotenterol. 2011;46:318‐324. 10.1007/s00535-010-0347-9 21103997

[76] Okada K , Suzuki S , Naito S , et al. Incidence of metachronous gastric cancer in patients whose primary gastric neoplasms were discovered after *Helicobacter pylori* eradication. Gastrointest Endosc. 2019;89:1152‐1159. 30825537 10.1016/j.gie.2019.02.026

[77] Yamaji Y , Watabe H , Yoshida H , et al. High‐risk population for gastric cancer development based on serum pepsinogen status and lifestyle factors. Helicobacter. 2009;14:81‐86. 19298334 10.1111/j.1523-5378.2009.00665.x

[78] Ohata H , Kitauchi S , Yoshimura N , et al. Progression of chronic atrophic gastritis associated with *Helicobacter pylori* infection increases risk of gastric cancer. Int J Cancer. 2004;109:138‐143. 14735480 10.1002/ijc.11680

[79] Esposito G , Pimentel‐Nunes P , Angeletti S , et al. Endoscopic grading of gastric intestinal metaplasia (EGGIM): a multicenter validation study. Endoscopy. 2019;51:515‐521. 30577062 10.1055/a-0808-3186

[80] Tran‐Duy A , Spaetgens B , Hoes AW , de Wit NJ , Stehouwer CD . Use of proton pump inhibitors and risks of fundic gland polyps and gastric cancer: systematic review and meta‐analysis. Clin Gastroenterol Hepatol. 2016;14:1706‐1719.e5. 27211501 10.1016/j.cgh.2016.05.018

[81] Poulsen AH , Christensen S , McLaughlin JK , et al. Proton pump inhibitors and risk of gastric cancer: a population‐based cohort study. Br J Cancer. 2009;100:1503‐1507. 19352380 10.1038/sj.bjc.6605024 PMC2694435

[82] Cheung KS , Chan EW , Wong AYS , Chen L , Wong ICK , Leung WK . Long‐term proton pump inhibitors and risk of gastric cancer development after treatment for *Helicobacter pylori*: a population‐based study. Gut. 2018;67:28‐35. 29089382 10.1136/gutjnl-2017-314605

[83] Pimentel‐Nunes P , Libanio D , Marcos‐Pinto R , et al. Management of epithelial precancerous conditions and lesions in the stomach (MAPS II): European Society of Gastrointestinal Endoscopy (ESGE), European Helicobacter and Microbiota Study Group (EHMSG), European Society of Pathology (ESP), and Sociedade Portuguesa de Endoscopia Digestiva (SPED) guideline update 2019. Endoscopy. 2019;51:365‐388. 30841008 10.1055/a-0859-1883

[84] Banks M , Graham D , Jansen M , et al. British Society of Gastroenterology guidelines on the diagnosis and management of patients at risk of gastric adenocarcinoma. Gut. 2019;68:1545‐1575. 31278206 10.1136/gutjnl-2018-318126 PMC6709778

[85] Kamada T , Haruma K , Ito M , et al. Time trends in *Helicobacter pylori* infection and atrophic gastritis over 40 years in Japan. Helicobacter. 2015;20:192‐198. 25581708 10.1111/hel.12193 PMC6905084

[86] Rugge M , Meggio A , Pravadelli C , et al. Gastritis staging in the endoscopic follow‐up for secondary prevention of gastric cancer: a 5‐year prospective study of 1755 patients. Gut. 2019;68:11‐17. 29306868 10.1136/gutjnl-2017-314600

